# A prospective evaluation of the role of Vascular Endothelial Growth Factor (VEGF) and the immune system in stage III/IV melanoma

**DOI:** 10.1186/s40064-015-0951-5

**Published:** 2015-04-17

**Authors:** Nicole Marie Agostino, Christine Saraceni, Hope Kincaid, Wenjing Shi, Wendy Kay Nevala, Svetomir Markovic, Suresh G Nair

**Affiliations:** Lehigh Valley Health Network, Department of Hematology Oncology, John and Dorothy Morgan Cancer Center, 1240 S. Cedar Crest Blvd, Suite 401, Allentown, PA 18103 USA; Mayo Clinic, Rochester, MN USA

**Keywords:** Melanoma, Stage IV, Biomarkers, VEGF, Tregs, TH1/TH2 ratio, CD4+/CD8+ ratio, LDH

## Abstract

**Background:**

The immune system and vascular endothelial growth factor (VEGF) may be influential in melanoma behavior. We performed a prospective, exploratory analysis in 10 stage III and 22 stage IV melanoma patients to observe factors influencing outcomes.

**Patients and methods:**

Patients accrued during 2010 and 2011 were treated according to standard protocols for disease stage. We analyzed selected biomarkers for predictive patterns of clinical response. Survival outcomes were calculated using Kaplan-Meier curves.

**Results:**

Baseline LDH was negatively correlated with length of survival and positively correlated to baseline VEGF in stage IV melanoma patients. We found a positive correlation between peripheral blood Treg concentrations and baseline VEGF in stage IV patients. No stage III patients died during the study period; median survival for stage IV patients was 48 months using a Kaplan-Meier survival curve, which illustrates the enrichment for exceptional stage IV survivors. Six stage IV patients remain disease free, including 4 of the 10 patients who received IL-2 +/− metastatectomy.

**Conclusions:**

Recent advances in immunotherapy have demonstrated durable therapeutic responses which may favorably impact survival. Examining T-cell characteristics of metastatic melanoma patients may gain further insight into underlying immunomodulation mechanisms to guide improved therapies.

## Introduction

As the incidence of cutaneous melanoma has risen incrementally in the United States, it has become a focus of public health concern. In 2014, an estimated 76,100 persons will be diagnosed with melanoma with 9,710 estimated deaths (American Cancer Society. Cancer Facts and Figures 2014). Survival rates are largely dependent on the stage of disease at initial diagnosis. Prior to 2011, treatment options have been limited and, overall, disappointing for stage IV melanoma. High-dose interleukin-2 (IL-2) as well as traditional chemotherapies comprised the mainstay of treatment. A new paradigm for primary systemic treatment for metastatic melanoma has shifted toward immunotherapy including IL-2, anti-CTLA4 monoclonal antibody (ipilimumab), and programmed cell death 1 antibody (anti-PD1), as well as targeted therapies (BRAF or MEK inhibition). As the preferred sequence of treatment in metastatic melanoma is yet undefined, it becomes important to delineate prognosticators of response with selected treatments.

High-dose IL-2 is associated with prolonged survival in selected individuals and is a potentially curative therapy in metastatic melanoma; hence, it deserves special consideration. In the metastatic setting, complete response rates approach 6% with partial response rates of approximately 10% (Atkins et al. 1999). Prior to initiation of therapy, there is currently no way to select patients who will favorably respond. Moreover, despite its durable efficacy, IL-2 induces a marked pro-inflammatory cascade responsible for its antitumor effect as well as systemic toxicities, including the systemic capillary leak syndrome. In an effort to better define characteristics of patients who may benefit from IL-2 therapy, Phan and colleagues (Phan et al. 2001) retrospectively reviewed their experience of 374 patients with stage IV melanoma who were treated with high-dose IL-2 from 1988 – 1999. Metastatic subset of disease was influential on response rates (RR), with cutaneous or subcutaneous metastatic disease having a RR of 53.6% compared to 12.4% for other sites of metastatic disease. A negative trend was noted for intracranial metastases. Other factors significantly related to response included post-treatment lymphocytosis and immunologic toxicity, such as vitiligo. Using gene microarray profiling, Sabatino and colleagues (Sabatino et al. 2009) identified that pre-treatment levels of vascular endothelial growth factor (VEGF) was an independent predictor of clinical response in IL-2 treated patients. They further defined that patients with VEGF levels >125 pg/mL exhibit significantly reduced overall survival (OS) (13 versus 23.3 months; p < 0.007) than patients below this cutoff.

Angiogenesis inhibition by targeting VEGF in combination with chemotherapy has been investigated in metastatic melanoma. High plasma VEGF levels have been associated with poor clinical outcomes in the setting of metastatic melanoma (Brychtova et al. 2008). The North Central Cancer Treatment Group conducted a phase 2 trial combining carboplatin, weekly paclitaxel, and biweekly bevacizumab in stage IV melanoma (Perez et al. 2009). Partial remission was achieved in 9 (17%) of the patients and stable disease achieved for at least 8 weeks in 30 (57%) of the patients. Median progression-free survival (PFS) and median OS were 6 months and 12 months, respectively. Plasma VEGF-A levels (the most common isoform) were measured pretreatment and at 4 weeks. There was no association between VEGF-A levels and PFS (Perez et al. 2009). Additionally, serum lactate dehydrogenase (LDH) is a well-recognized independent prognostic indicator for survival in patients with metastatic melanoma (Sirott et al. 1993, Agarwala et al. 2009, Abeloff et al. 2008) and is a surrogate marker for disease progression in stage IV disease (Agarwala et al. 2009).

Immunotherapies have emerged as an area of active focus in melanoma research. The immune system produces various CD4+ T-cell responses including Th1 and Th2. A distinct subset of CD4+ T-cells were identified in 2005 called Th17. These T helper cells regulate host defense and may have a role in tumor immunity (Paulos et al. 2014). We limited our analyses to the Th1 and Th2 subsets. The Th1 response is characterized by a dominance of the cytokine interferon-γ, which stimulates CD8+ cytotoxic T-lymphocytes that have an antineoplastic effect. Th2 responses are dominated by the cytokine interleukin-4 (IL-4) and are thought to be pro-neoplastic and nurture a chronic inflammatory state (Abeloff et al. 2008; Jiang & Chess 2006). Chronic inflammation has been linked to the progression of melanoma at the level of the tumor microenvironment with associated immune dysregulation (Nevala et al. 2009).

Hernberg and colleagues (Hernberg et al. 2004) observed that an early increase in CD4+/CD8+ ratio in metastatic melanoma patients during treatment was a favorable prognostic indicator. In Hernberg’s study, patients who had decreasing levels of CD8+ T-cells had a median survival of 27.8 months; whereas, patients with increasing levels had a median survival of 10.8 months. Cytokine-secreting regulatory T-cells (Tregs) mediate regulatory function of immune response and can shift immune system balance toward Th1 or Th2 dominant state (Jiang & Chess 2006). IL-2 is a T-cell growth factor and mediates Treg expansion *in vivo*, favoring a Th2-dominated state, which may account for patterns of failure with IL-2 therapy. It has been suggested that selective inhibition of IL-2-mediated enhancement of Tregs may improve therapeutic efficacy of IL-2 (Ahmadzadeh & Rosenberg 2006). Nevala and colleagues (Nevala et al. 2009) demonstrated that malignant melanocytes highly up-regulate VEGF transcripts as measured by plasma VEGF concentrations and perpetuate a chronically Th2-mediated state.

Our study was a hypothesis-generating, prospective evaluation of the role of VEGF and selected immune-modulatory cells including Treg, Th1/Th2 ratio and CD4+/CD8+ ratio, as well as BRAF status and baseline LDH in stage III and IV melanoma patients undergoing active treatment. Our goal was to observe trends that may help guide initial choice of therapy in advanced melanoma or reveal immunomodulary patterns. After an extensive review of the literature, we found no studies on the correlation among LDH, Tregs, VEGF or Th1/Th2 ratio in relation to each other.

## Patients and methods

### Patients and treatment

We prospectively gathered data and collected blood samples via venopuncture from 10 stage III and 22 stage IV patients referred to our institution for the treatment of melanoma. At study enrollment, we collected 90 mls of blood as drawn into acid citrate dextrose vacutainers and PBMCs were isolated using a Ficoll gradient. Plasma was also collected prior to the ficoll gradient and stored at −80°C in 1 ml aliquots. Isolated PBMCs were washed with 1x PBS and suspended in freezing media (90% Cosmic Calf serum and 10% DMSO). PBMCs were stored and batch analyzed by flow cytometer. Patient characteristics are described in Table 1. Prior therapies are listed in Table 2. The Lehigh Valley Health Network Institutional Review Board approved the study before accrual of any study participants. All study participants provided written informed consent. The patients were treated according to standard protocols for disease stage including resection and interferon or observation for stage III patients and resection +/− GM-CSF, IL-2, ipilimumab, vemurafenib, carboplatin/paclitaxel/bevacizumab and temozolomide for stage IV patients. Original biopsy specimens were sent for BRAF mutational analysis. Some patients received treatments for melanoma prior to enrollment.

**Table 1:** **Clinical and demographic characteristics of patients**

	**Entire sample (N = 32)**	**Stage IV(N = 22)**	**Stage III(N = 10)**
Age (Mean ± SD)	56.44 ± 10.52	55.77 ± 11.10	57.90 ± 9.72
Males N (%)	22 (68.8)	15 (68.2)	7 (70.0)
Stage IV Sub-type N (%)			
IVa	-	2 (9.1)	-
IVb	-	3 (13.6)	-
IVc	-	17 (77.3)	-
BRAF			
Mutated	11 (34.4)	7 (31.8)	4 (40)
Wild Type	16 (50)	14 (63.6)	2 (20)
Not Available	5 (15.6)	1 (4.5)	4 (40)
Length of follow-up in months (Mean ± SD)	39.28 ± 21.86	36.32 ± 24.63	45.80 ± 12.73

BRAF, B-type Raf kinase; SD, standard deviation.

**Table 2:** **Treatments for stage III and stage IV patients**

**Stage III (n = 10)**		
	**Treatments**	**# patients**
	None	3
	Immunotherapy	7
**Stage IV (n = 22)**		
	**Treatments**	**# patients**
	Immunotherapy	11
	Chemo	2
	Immunotherapy, Chemo	3
	Immunotherapy, Chemo, Anti-angiogenic	3
	Vaccine, Immunotherapy	1
	Immunotherapy, Chemo, Anti-angiogenic, Targeted	2

Chemo – chemotherapy, Targeted – targeted therapy, Immunotherapies include: High-dose Interleukin-2, Ipilimumab, granulocyte macrophage colony-stimulating factor, and interferon; Chemotherapies include: carboplatin, paclitaxel, paclitaxel nanoparticle albumin-bound, and temozolomide; Targeted therapies include: vemurafenib; Anti-angiogenic agents include: thalidomide, bevacizumab.

Subject inclusion criteria included age >18 years old, histological confirmation of stage III or IV melanoma as defined by the AJCC Staging Handbook (Edge et al. 2010), ability to provide informed consent, and willingness to participate. Exclusion criteria included conditions associated with VEGF level elevations such as chronic non-healing wounds, uncontrolled hypertension defined as blood pressure >140/90 on 3 separate measurements on 3 separate days, and uncontrolled on antihypertensive therapy; uncontrolled inflammatory state (connective tissue disease, inflammatory bowel disease, chronic infections, or active, second malignancy); and pregnancy.

All serum samples were prospectively collected and included baseline LDH and baseline VEGF levels with VEGF-A isoform (Table 3). The VEGF-A ELISA was performed at room temperature per procedural guidelines of R & D systems (R & D Systems, Minneapolis, MN). The plate was read at 450 nm and 540 nm on a luminescence microplate reader. The wavelength correction was done by subtracting the reading of 540 nm from that of 450 nm. Lactate dehydrogenase levels were assayed in a commercial laboratory (Health Network Labs, Allentown, PA) on a Siemen’s Dimension Vista 500 (Siemens Medical Solutions, Malvern, PA). Substrate L-lactate was buffered at a pH of 9.4 and lactate dehydrogenase was added to that substrate to yield pyruvate and NADH. This absorbs light at 340 nm allowing rate reaction measurement. BRAF V600E testing was performed by Clarient laboratories (Clarient Inc, Aliso Viejo, CA) by allele-specific polymerase chain reaction.

**Table 3:** **Serum measurements**

	**Entire group(N = 32)**	**Stage IV(N = 22)**	**Stage III(N = 10)**
LDH U/L (Median, Q1, Q3)	223.50, 199.25, 263.75	230.00, 211.50, 307.50	200.00, 171.50, 245.50
VEGF pg/mL (Mean ± SD)	400.40 ± 252.88	422.69 ± 294.45	351.37 ± 118.71
Treg % (Mean ± SD)	27.13 ± 6.15	27.56 ± 6.43	26.18 ± 5.68
Th1/Th2 (Mean ± SD)	0.62 ± 0.17	0.64 ± 0.15	0.58 ± 0.20
CD4/CD8 (Median, Q1, Q3)	1.70, 1.22, 2.14	1.76, 1.01, 2.19	1.65, 1.32, 2.05

LDH, lactic acid dehydrogenase; Q1, first quartile; Q3, third quartile; VEGF, vascular endothelial growth factor; SD, standard deviation; Treg, regulatory T cell.

Table 2: Q1 = first quartile, Q3 = third quartile, SD – standard deviation.

**Figure 1:**
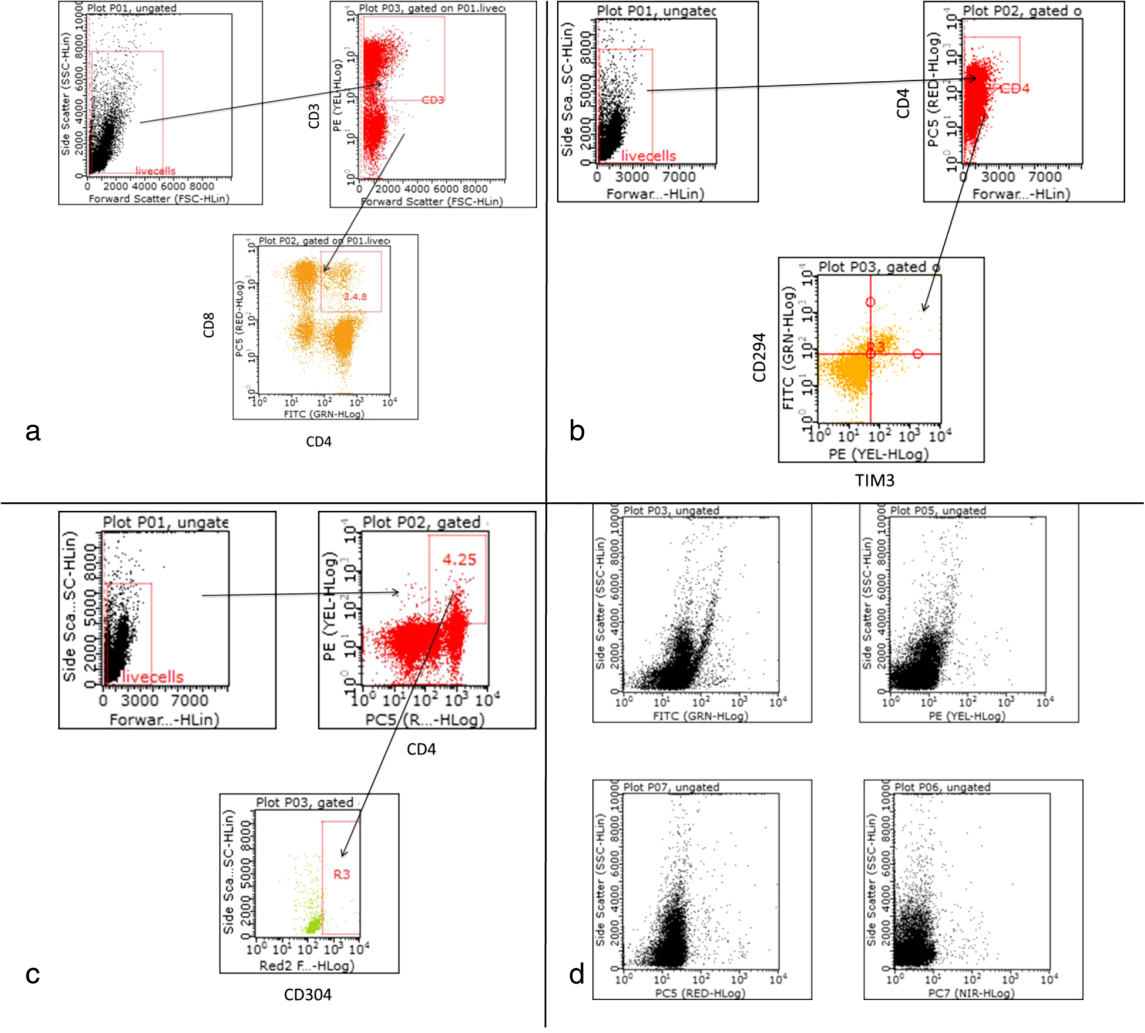
Scatterplots of Gating Analyses for CD4+, CD8+, Th1, Th2 and Treg with isotype controls, **(1a)** - CD4+ and CD8+, **(1b)** - Th1 (CD4+ and TIM3+), Th2 (CD4+ and CD294+), **(1c)** - Treg (CD4+, CD25+, and CD304+), **(1d)** -Isotype Controls.

Baseline direct measurements of Th1 and Th2 were performed by flow cytometry. Cellular phenotypes were determined using multiparametric flow cytometry. The antibodies used are as follows: FITC anti-human CD294 and PE anti-human TIM-3 (R & D Systems, Minneapolis, MN), FITC anti-human CD3, PC5 anti-human CD4 and PC7 anti-human CD8, FITC anti-human CD16, PE anti-human CD56, PC5 anti-human CD3, FITC anti-human CD69, PE anti-huamn CD62L, FITC anti-human CD14, PC5 anti-human CD11c, PE anti-human HLA-DR, PC7 anti-human CD80, PC7 anti-human CD83, PC7 anti-human CD86, PC7 anti-human CD123, PE anti-CD40, FITC anti-human CD25, and PE anti-human CD304 (neuropilin-1 receptor) (BD Pharmingen, Piscataway, NJ) Previously viably frozen cells were thawed and stained with surface antibodies for 30 minutes at 4°C in FACS buffer (PBS with 0.5% BSA and 0.05% Sodium Azide). After incubation all cells were washed twice in FACS buffer and suspended in 4% paraformaldehyde in PBS and data was collected on a Gauva 8HT flow cytometer Millipore Billerica, MA) and; data analysis was performed using FlowJo software (FlowJo Ashland, OR). T-helper 1 cells were defined as CD4+ and TIM3+ while T-helper 2 cells were CD4+ and CD294+. Our gating strategies using Tim3+ to define Th1 and CD294+ to define Th2 subsets have been previously described (Lee et al. 2011; Monney et al. 2001). Lee et al. (2011) have described the same CD4+/CD294+ gating strategy we used in our study. Monney et al. (2001) have characterized Tim-3 expression on Th1 cells. The percentages are of the live-gated cells on forward and side scatter dot plots. The Th1:Th2 ratios were calculated by dividing the percentage of Th1 positive cells by the percentage of Th2 positive cells (Table 3). In this study Treg cells were defined as CD4+, CD25+ and CD304+ triple positive cells. The percentage of cells was determined from CD4+ cells from the live gate. From the CD4+ gate, the percentage of CD25+ and CD304+ cells were determined. Although neuropilin-1 has been identified as a specific marker of murine Treg its applicability as a marker of human Treg has been questioned (Milpied et al. 2009). However, CD304+ has been previously described as a marker for Tregs by others (Battaglia et al. 2010; Piechnik et al. 2013). Examples of gating strategies (Figures 1a, 1b, and 1c) and isotype controls (Figure 1d) are presented in Figure 1.

### Statistical analyses

This study involved a convenience sample of 32 melanoma patients seen at our institution between 2010 and 2011. We assessed the correlation between VEGF, several immune-modulatory cells and cytokines, BRAF status, baseline LDH, survival, and length of survival. Pearson’s Correlation Coefficient (r) was used when both variables were continuous and normally distributed. Spearman’s Rank Correlation Coefficient (r_s_) was used if both variables were continuous and one or both were not normally distributed. The Point-Biserial Correlation Coefficient (r_pb_) was reported if one of the variables was dichotomous and the other was numeric. The Phi Coefficient was reported if both of the variables were dichotomous (Warner 2008). The criteria set forth by Cohen (1988) were used to interpret the correlation coefficients. A correlation coefficient of 0.1 is considered “small”, 0.3 is considered “medium”, and 0.5 is considered “large”. Normality of the variables was determined using the skewness statistic as well as the Shapiro-Wilk Test, as this test is appropriate for sample sizes <50 (Lund Research Ltd. 2013). Kaplan-Meier Curves were generated to illustrate median survival in stage IV patients with low baseline LDH (defined as <250) compared to those with high baseline LDH (defined as > =250). A Cox Proportional Hazards Regression model was used to assess whether or not any of the variables that were found to be correlated with survival were also predictive of survival.

Data was analyzed separately for stage III and IV patients. The analysis was conducted using IBM® SPSS® Statistics version 22.0 and MedCalc® version 12.4.0.0. All tests were two-tailed. Alpha was set at 0.05.

## Results

There were 22 stage IV and 10 stage III melanoma patients included in this study. The mean length of follow-up was 45.80 (±12.73) months for stage III patients and 36.32 (±24.63) months for stage IV patients (Table 1). At study completion, none (0.0%) of the stage III patients and 13 (59.1%) of the stage IV patients had expired.

**Figure 2:**
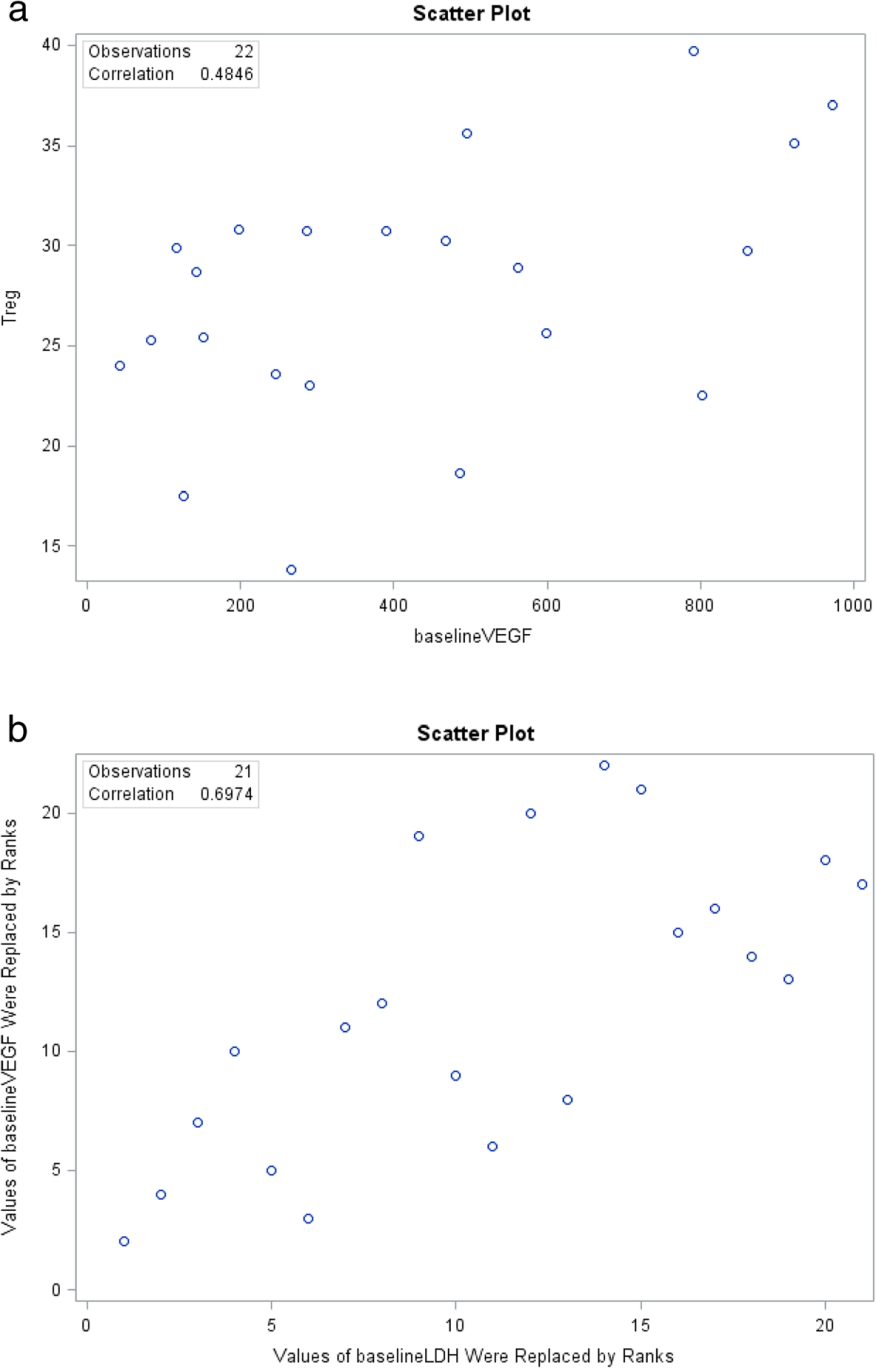
Scatter Plots of Correlation. **a** between Baseline VEGF and Baseline Treg in Stage IV Patients **b** between Baseline VEGF and Baseline LDH in Stage IV Patients.

**Figure 3:**
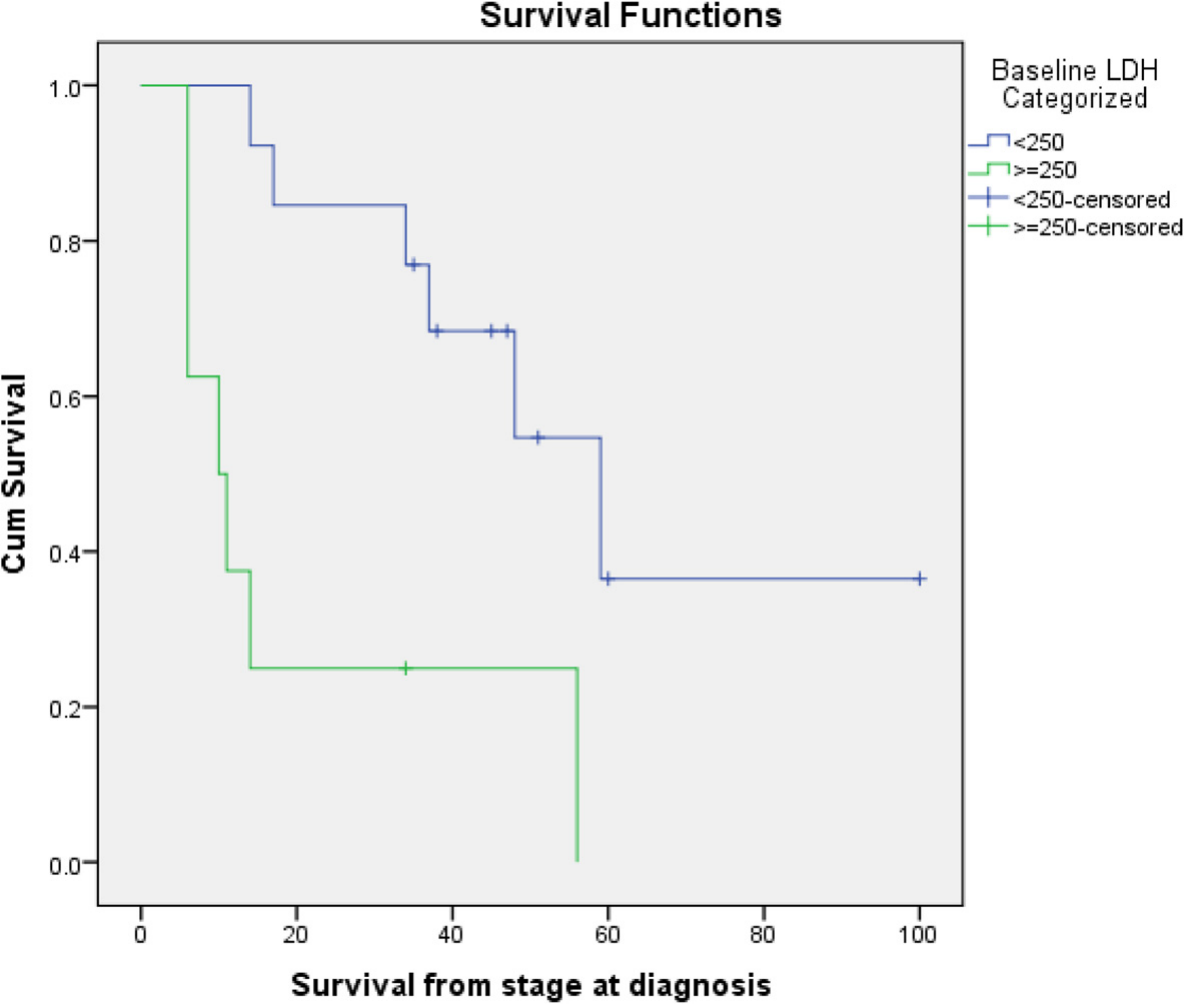
Survival Curves for Stage IV Patients Based on LDH.

In stage IV patients, we found a medium, positive correlation *(r = .485, n = 22, p = .022)* between Treg levels and baseline VEGF (Figure 2a), and a large, positive correlation *(r*_*s*_ *= .697, n = 21, p < .001)* between baseline LDH and VEGF (Figure 2b). There was a medium, negative correlation *(r*_*s*_ *= −.495, n = 21, p = .022)* between baseline LDH and length of survival. Median survival for stage IV patients was 48 months. Median survival could not be calculated for stage III patients, who were all still alive at study completion. Median survival for stage IV patients with low baseline LDH and those with high baseline LDH was 59 months and 10 months, respectively (Figure 3). Baseline LDH was found to be predictive of survival in stage IV patients (HR = 1.0017, 95% CI: 1.0003 – 1.0032, p = 0.0214).

## Discussion

The landscape of metastatic melanoma treatment has evolved strikingly in recent years with the development of antitumor molecular targets, including BRAF-inhibitors such as vemurafinib and dabrafenib (Flaherty et al. 2010a) and MEK-inhibition with trametinib (Flaherty et al. 2010b). Ipilimumab, a fully human monoclonal antibody has been shown to block cytotoxic T-lymphocyte-associated antigen 4 (CTLA-4), an immune checkpoint molecule that down-regulates pathways of T-cell activation (Hodi et al. 2010). Newer therapies include monoclonal antibodies directed against the programmed death 1 (PD-1) checkpoint molecule (Topalian et al. 2012) or its ligand (PDL-1) (Brahmer et al. 2012). Each drug has a unique mechanism of action and newer therapies have a substantial financial burden. As oncology practice is evolving into a personalized-medicine treatment approach, it becomes important to define variables and patient characteristics in advanced disease that may be predictive of treatment response, or aid in selection of the patients most likely to benefit as well as in selection of optimal treatments.

Angiogenesis, the formation and differentiation of blood vessels, is fundamental in neoplastic growth and metastatic dissemination (Folkman 1995). VEGF has been recognized as an important regulator of pathologic angiogenesis and is associated with tumor progression and poor outcomes in a variety of human cancers including metastatic melanoma (Brychtova et al. 2008; Salven et al. 1997; Gasparini et al. 1997). Measurement of serum VEGF has been utilized as a surrogate marker of tumor angiogenesis (Kraft et al. 1999) and as a predictive biomarker for prognosis and therapeutic response (Sabatino et al. 2009). Disruption in immune homeostasis with a shift toward a Th2-dominant or chronic inflammatory state by tumor-derived VEGF has been previously reported. It is this ‘reprogramming’ of systemic immunity that may be permissive to tumorigenesis and metastatic propensity (Nevala et al. 2009). Malignancy-related immunosuppression in conjunction with angiogenesis is active in metastatic melanoma.

We formulated a hypothesis-generating pilot study utilizing a practical means of measuring levels of immunosuppression and angiogenesis in melanoma. We examined the relationship between VEGF and biomarkers of immune function, including Tregs, Th1/Th2 ratio and CD4+/CD8+ ratio, in a prospective cohort of patients. Our observations may be useful as a basis for planning of larger trials in the future. To our knowledge, we observed for the first time a positive relationship between baseline VEGF and Tregs as well as a positive correlation between baseline VEGF and LDH in stage IV patients. We found no association between baseline VEGF, BRAF status and CD4+/CD8+ ratio with survival. We sought to examine the impact of baseline measurable markers of immune status on survival in stage IV melanoma patients treated aggressively at a high-dose IL-2 center during the time period of 2010 and later. Our general treatment philosophy for stage IV disease was immunotherapy as first-line, followed by targeted therapy and chemotherapy as later options. This is aligned with the 2013 Society for Immunotherapy of Cancer consensus statement (Kaufman et al. 2013).

These pilot results suggest a link between angiogenesis activation (measured by serum VEGF) and possible development of immune tolerance (measured by Treg frequency). Regulatory T cells (T regs) influence antitumor responses by their potent immunosuppressive function. High concentrations of T regs within tumors as well as peripheral blood in various cancer subtypes have been linked to poorer prognosis (Nishikawa & Sakaguchi 2010). Their relationship to VEGF continues to be investigated particularly the relationship within tumor tissue (tumor infiltration). VEGF has been studied as a promoter of Treg activation in antitumor immunity. Currently, it is favored that immune evasion is more correlated with expansion of Tregs within the tumor microenvironment rather than in the peripheral blood. In a murine in vitro model, Hansen (Hansen 2013) describes a neuropilin1/VEGF – mediated trafficking of Tregs as a prerequisite for tumor infiltration. Our study suggests a potential correlation between serum VEGF expression and T reg expansion in peripheral blood in melanoma patients. Ongoing investigation is needed to fully elucidate the role of VEGF in regulatory T cell functions.

LDH was the only baseline parameter to correlate with survival. Our model found that baseline LDH was predictive of survival in stage IV patients, which means that with each one-unit increase in LDH, estimated mortality risk increases by 0.17% and for a 100-unit increase in LDH, estimated mortality risk increases by 18.5%. In our study patients with higher LDH levels have higher VEGF levels, an association which has been previously reported by Sabatino et al. (Sabatino et al. 2009). In this prior report, elevations of VEGF were associated with significantly reduced overall survival (Sabatino et al. 2009). Our preliminary observations may warrant a larger prospective study examining LDH and serum VEGF levels in patients being considered for high-dose IL-2 therapy. Elevation of VEGF and reduced overall survival did not reach statistical significance in our study. However, there was a trend toward significance. For stage IV patients the correlation between survival at stage from diagnosis and baseline VEGF was −0.375 (p-value = 0.085). Therefore, baseline VEGF and survival were negatively correlated. If our study population were larger, this observation may have reached statistical significance. These baseline serum (LDH and VEGF) and cellular (Treg and Th1/Th2) parameters are worthy of further study in new treatment paradigms, which are rapidly developing with the era of new immunotherapies. Tumor biopsies prior to and during treatment are crucial to investigate local changes around the tumor milieu with ipilimumab and anti-PD1 therapies, especially with regard to Treg around tumor. Systemic Treg and tumor Treg may not necessarily correlate except for extreme cases, and the local effect within the tumor microenvironment is likely to be of greater importance than systemic Treg levels. Study of these crucial parameters may help us define the optimal options to achieve best survival. At present, studies are ongoing or initiating combining IL-2 with anti-PD1, ipilimumab with anti-PD1, sequential IL-2/ipilimumab/anti-PD-1, sequential ipilimumab/anti-PD1, among others. This may establish treatment paradigms, based on baseline immune status and angiogenesis activation, that are more efficacious and cost-effective.

There are several limitations to our study which need to be mentioned. First, we used a convenience sampling method as opposed to a random sampling method. Second, both newly diagnosed stage IV melanoma patients and exceptional survivors of stage IV melanoma, many who had received past immunotherapy treatments, were included. Therefore, it is possible that our sample may not accurately represent the larger population of all stage III and IV melanoma patients as is reflected in our exceptionally long overall survival. Nevertheless, examining T-cell characteristics of metastatic melanoma patients, including exceptional melanoma survivors, may gain us further insight into underlying immunomodulation mechanisms that may help guide improved therapies. An additional limitation of our study is the small sample size, which limited our ability to collect and control for confounding factors in the regression analysis. The prospective collection of data contributes to the strength of our study, as it ensures the accuracy of the observations. Future prospective studies, with larger sample sizes, are needed to further validate our observations.
